# P-1708. Frontline Febrile Neutropenia Antibiotic Treatment Across Institution Types: A Multicenter Survey Study

**DOI:** 10.1093/ofid/ofae631.1874

**Published:** 2025-01-29

**Authors:** Maia Castillo, Derick Gross, Stephanie Harding

**Affiliations:** Wesley Medical Center, Boise, Idaho; Wesley Medical Center, Boise, Idaho; Wesley Medical Center, Boise, Idaho

## Abstract

**Background:**

American Society of Clinical Oncology febrile neutropenia guidelines highlight the importance of quick empiric antipseudomonal coverage, and use of gram-positive active agents only in special circumstances. Despite this, first line vancomycin use increased over the years, thus, our study hoped to gain information on how institutions treat febrile neutropenia and if any patterns emerge between institution types.

**Methods:**

A survey was distributed via email to IDSA and ACCP listservs. A survey tool was used for survey questions and to collect responses pertaining to febrile neutropenia management, institution demographics, and routine antibiotics used. Descriptive statistics, chi-squared tests and Mann-Whitney U tests were used to analyze the data as indicated.

**Results:**

Eighty-four survey results were included in the analysis. When comparing academic medical centers (AMC) to non-AMCs there was more initial oncology responsibility for treatment (50.9% vs 22.6%; p-value = 0.047) and less empiric MRSA coverage (p-value = 0.039). When comparing institutions with > 500 beds to those with < = 500 beds similar results were seen with more initial oncology responsibility (53.2% vs 25%; p-value = 0.047), and more primary oncology responsibility in larger locations (70.2% vs 36.1%; p-value = 0.039), and less empiric MRSA coverage (p-value = 0.02). When comparing institutions with internal protocols versus without, there was less empiric MRSA coverage with a protocol (p-value = 0.006). No statistically significant difference in percentage of patients treated with MRSA or pseudomonal coverage were found when comparing service lines responsible for febrile neutropenia treatment.

**Conclusion:**

Institution size or academic affiliation does not directly impact adherence to guidelines, internally published FN guidelines can increase adherence to IDSA/ASCO guidelines and are a modification that any institution can implement. Having a small sample size with perception-based data was a large limitation of this study, therefore future studies with larger sample sizes and concrete usage data could be pursued in the future to better understand febrile neutropenia treatment guideline adherence.

**Disclosures:**

**All Authors**: No reported disclosures

